# Eight complete and four draft genome sequences of nonpathogenic *Avibacterium paragallinarum* isolates from naive, healthy layer chickens in the USA

**DOI:** 10.1128/mra.01334-24

**Published:** 2025-08-15

**Authors:** Mostafa M. S. Shelkamy, Amro Hashish, Mariela E. Srednik, Eman Gadu, Maria Chaves, Nubia Macedo, Qijing Zhang, Yuko Sato, Stephan Schmitz-Esser, Mohamed El-Gazzar

**Affiliations:** 1 Department of Veterinary Diagnostic and Production Animal Medicine, College of Veterinary Medicine, Iowa State University 1177, Ames, Iowa, USA; 2 Department of Avian and Rabbit Medicine, Faculty of Veterinary Medicine, Suez Canal University 68831, Ismailia, Egypt; 3 National Laboratory for Veterinary Quality Control on Poultry Production, Animal Health Research Institute, Agriculture Research Center 357113, Giza, Egypt; 4 Department of Veterinary Sciences, University of Wyoming 4416, Laramie, Wyoming, USA; 5 Department of Avian and Rabbit Diseases, Faculty of Veterinary Medicine, Mansoura University 68779, Mansoura, Egypt; 6 Department of Veterinary Microbiology and Preventive Medicine, College of Veterinary Medicine, Iowa State University 1177, Ames, Iowa, USA; 7 Department of Animal Science, Iowa State University 1177, Ames, Iowa, USA; Loyola University Chicago, Chicago, Illinois, USA

**Keywords:** *Avibacterium paragallinarum*, nonpathogenic, naive, healthy layer chickens, infectious coryza

## Abstract

*Avibacterium paragallinarum* is a primary bacterial pathogen causing infectious coryza (IC), a respiratory disease of chickens. However, nonpathogenic *Avibacterium paragallinarum* (npAP) has been discovered in naive, healthy chickens, complicating IC diagnosis. Here, we report eight complete and four draft genome sequences of npAP isolates from four US states.

## ANNOUNCEMENT

*Avibacterium paragallinarum* (AP) is the causative agent of infectious coryza (IC) in chickens, leading to significant economic losses in the poultry industry (1). Recently, genetically divergent nonpathogenic AP (npAP) isolates, which still fall within the AP species, were identified in naive, healthy layer (NHL) flocks (2, 3). npAP is prevalent in the US NHL flocks, complicating the diagnosis of IC (4).

In this study, bacterial isolates were obtained from the infraorbital sinuses of NHL chickens (5) using selective media consisting of Mueller-Hinton agar, fetal bovine serum, nicotinamide adenine dinucleotide, and inhibitors (6). Streaked plates were incubated for 48 hours at 37°C with 5.2% CO_2_, and AP colonies were confirmed by MALDI-TOF (7) and the *recN* qPCR assay (8).

For Illumina sequencing, bacterial DNA was extracted using the KingFisher Flex machine and the MagMAX Pathogen RNA/DNA Kit (Thermo Fisher Scientific, Waltham, MA, USA). Sequencing libraries were prepared using different kits (Table 1) to generate 2 × 250 bp or 2 × 300 bp paired-end reads and sequenced on the Illumina MiSeq system (Illumina, San Diego, CA, USA). For Oxford Nanopore Technology (ONT) sequencing, genomic DNA extraction was performed using the Genomic-tip 20/G kit (Qiagen, Germany). ONT libraries were prepared using the Ligation Sequencing Kit (SQK-LSK109), barcoded with the Native Barcoding Expansion (EXP-NBD114), and DNA fragments of 3 kb or longer were enriched by Long Fragment Buffer from ONT (Oxford, UK). Sequencing was conducted on a MinION Mk1B flow cell R9.4.1 (FLO-MIN106) for 72 hours and monitored via MinKNOW (version 23.04.6). Post-run base calling was performed with Guppy (version 6.5.7) with super high accuracy mode enabled.

**TABLE 1:** Metadata, sequencing, and genomic characteristics of 12 npAP isolates^*a*^

Metadata	Isolate name	npAP/OH-USA/20230213/S1-2	npAP/OH-USA/20230213/S1-3	npAP/OH-USA/20230213/S1-4	npAP/GA-USA/20231216/S1-1	npAP/GA-USA/20231216/S1-2	npAP/GA-USA/20231220/S2-1	npAP/GA-USA/20231220/S2-3	npAP/NC-USA/20240110/S1-1	npAP/GA-USA/20231220/S2-2	npAP/GA-USA/20231220/S2-4	npAP/OH-USA/20230315/S2-1	npAP/FL-USA/20231025/S1-1
	Geographic location	Ohio	Ohio	Ohio	Georgia	Georgia	Georgia	Georgia	North Carolina	Georgia	Georgia	Ohio	Florida
	Production system	MAC	MAC	MAC	SFMH	SFMH	SFMH	SFMH	SFMH	SFMH	SFMH	MAC	MAC
	Age (weeks)	44	NA^*j*^	NA	74	74	70	70	25	70	70	NA	83
Raw data	Number of Illumina MiSeq reads	922,464	939,212	697,976	5,395,178	580,108	4,484,802	1,131,752	4,835,104	6,055,496	945,750	739,250	637,394
	Illumina paired-end read length (nt)	300^*b*^	300^*b*^	300^*b*^	250^*c*^	300^*b*^	250^*c*^	250^*b*^	250^*c*^	250^*c*^	250^*b*^	250^*b*^	250^*b*^
	Total Illumina MiSeq sequencing data (Mbp)	209.02	212.86	179.69	1,348.79	132.22	1,121.2	284.07	1,208.78	1,513.87	237.38	185.55	124.48
	Number of ONT reads	504,191	124,034	229,750	456,668	1,225,813	396,973	601,888	388,581	525,719	753,819	1,110,720	7,837
	ONT read *N*_50_ (bp)	12,812	16,819	8,754	9,128	4,047	11,950	5,311	12,359	10,952	3,931	1,176	1,776
	Total ONT data (Mbp)	1,765.31	584.92	749.24	1,194.22	2,582.4	1,023.5	2,322.6	1,308.62	1,016.88	1,413.1	892.58	7.84
Assembly	Assembler	Flye version 2.9.1	Flye version 2.9.1	Flye version 2.9.1	Flye version 2.9.1	Flye version 2.9.1	Flye version 2.9.1	Flye version 2.9.1	Flye version 2.9.1	Flye version 2.9.1	Flye version 2.9.1	Unicycler version 0.4.8	Unicycler version 0.4.8
	Number of contigs	1	1	1	1	1	1	1	1	3	3	34	213
	CheckM completeness (%)^*d*^	99.3	99	99	99.4	98.8	99.3	98.9	99.5	99.3	98.9	98.4	98
	CheckM contamination (%)^*d*^	1.3	1.1	1.1	0.9	0.8	1.1	1.1	0.9	1	1.2	1.8	1
	GC content (%)	41.07	41	40.99	41.04	41	41.07	41.14	41.1	41.08	41.23	41.15	41.51
	Chromosome size (bp)	2,423,524	2,425,817	2,419,151	2,521,590	2,506,457	2,517,499	2,532,146	2,464,160	2,566,703	2,611,098	2,417,242	2,203,106
	Number of putative plasmids^*e*^ (bp)	0	0	0	1 (2,984)	0	0	0	1 (15,332)	0	0	*	*
	Average short read coverage (×)	79.22	79.81	69.06	152	47.4	170.1	85.24	167.04	182.99	70.56	52.35	51.04
	Average long read coverage (×)	183.69	178.76	182.52	467.44	205.3	207.6	201.86	521.73	207.07	194.14	167.15	9.33
	Contig *N*_50_	2,423,524	2,425,817	2,419,151	2,521,590	2,506,457	2,517,499	2,532,146	2,464,160	2,268,408	1,955,296	176,515	19,604
	Contig *L*_50_	1	1	1	1	1	1	1	1	1	1	5	37
Chromosome annotation	Number of CDSs	2,240	2,239	2,232	2,401	2,377	2,368	2,416	2,315	2,443	2,528	2,291	2,097
	Number of tRNAs	57	58	58	58	58	57	59	57	57	59	50	31
	Number of rRNAs	19	19	19	19	19	19	19	19	19	19	8	5
Genetic features	ANI^*f*^ score	96.70%	96.67%	96.66%	96.60%	96.56%	96.57%	96.54%	96.68%	96.59%	96.56%	96.39%	96.80%
	*HMTp210*^*g*^ (bp)	16,644	16,299	16,299	12,876	16,950	20,025	16,725	20,046	20,025	16,725	18,327	18,102
	*hctA^h^*	−	−	−	+	+	+	−	−	+	−	−	−
	Catalase	+	+	+	−	−	−	−	−	−	−	−	−
	RTX pre-toxin (*AvxA*)	−	−	−	−	−	−	−	−	−	−	+	+
GenBank data	BioSample accession number	SAMN44459154	SAMN44459155	SAMN44459156	SAMN44459159	SAMN44459160	SAMN44459161	SAMN44459163	SAMN44459165	SAMN44459162	SAMN44459164	SAMN44459157	SAMN44459158
	Assembled chromosome accession number	CP173233	CP173232	CP173231	CP173236	CP173230	CP173229	CP173228	CP173234	JBJBHD000000000	JBJBHC000000000	JBJBHF000000000	JBJBHE000000000
	Assembled Putative plasmid accession number				CP173237				CP173235				
	Illumina SRA accession	SRX26505944	SRX26505945	SRX26505948	SRX26505951	SRX26505952	SRX26505953	SRX26505955	SRX26505947	SRX26505954	SRX26505946	SRX26505949	SRX26505950
	ONT SRA accession	SRX26521028	SRX26521029	SRX26521030	SRX26521033	SRX26523164	SRX26523165	SRX26523167	SRX26523169	SRX26523166	SRX26523168	SRX26521031	SRX26521032
	The closest 16S rRNA gene match^*i*^	NR_042932.1	NR_042932.1	NR_044750.1	NR_042932.1	NR_042932.1	NR_042932.1	NR_042932.1	NR_042932.1	NR_042932.1	NR_042932.1	NR_042932.1	NR_042932.1

^*a*^ Naming format for non-pathogenic *Avibacterium paragallinarum* isolates: npAP/location/YearMonthDay/site-isolate number. MAC, multi-age layer complex; SFMH, single-flock-multi-house; *, unable to determine, small circular contigs that could be plasmids were identified but did not yield any hits in the PlasmidFinder database or NCBI BLAST. −, absent; +, present.

^*b*^ Illumina sequencing libraries prepared using Nextera XT DNA Library Prep Kit (Illumina, USA).

^*c*^ Illumina sequencing libraries prepared using NEBNext Ultra II FS DNA Library Prep Kit (New England Biolabs).

^*d*^ CheckM completeness percentage and CheckM contamination percentage were assessed according to reference 9.

^*e*^ Putative plasmids were identified based on the bandage plot.

^*f*^ The average nucleotide identity (ANI) percentage to the AP type strain NCTC11296 was calculated based on MUMmer via the JSpeciesWS service (10).

^*g*^ *HMTp210* gene of pathogenic AP is approximately 6,100 bp.

^*h*^ *hctA* is one of the capsular export genes; isolates with *hctA* possess the capsular polysaccharide locus.

^*i*^ The closest 16S rRNA gene match was determined using NCBI BLAST against rRNA/ITS databases with the selection of 16S ribosomal RNA sequences (Bacteria and Archaea).

^*j*^ NA, Non Available Data.

Default parameters were used for all software tools and web-based platforms unless otherwise indicated. Trim Galore version 0.6.5 (11) was used to trim low-quality bases and remove sequencing adaptors from the Illumina reads. The hybrid assembler Unicycler version 0.4.8 (12) was used to assemble two genomes, while the long-read assembler Flye version 2.9.1 (13) followed by two rounds of polishing with Illumina reads using Pilon version 1.23 (14) was used for the remaining 10 genomes (Table 1). The selection of either assembler was based on assembly quality as well as the quality and quantity of long-read data generated. Bandage plot version 0.8.1 (15) was used to check the circularity of chromosomes, and Quast version 5.2 (16) was used to assess assembly quality. All genomes were annotated using the RASTtk (17). These tools were accessed through BV-BRC version 3.35.5 (18). The publicly available assemblies in GenBank were annotated via PGAP version 6.8 (9).

While all 12 genomes were classified as npAP, they exhibited notable variability in key genes. For instance, four isolates are encapsulated (*hctA*+), whereas the others are not. Additionally, three npAP isolates display catalase positivity, differing from the typical biochemical activity of AP species. Two distinct putative plasmids were identified. Despite this variability, the lengthy *HMTp210* gene, ranging from 12,876 to 20,046 bp due to unique insertions, remains a defining feature of npAP. A summary of data is shown in Table 1.

## Data Availability

The genomes are available from NCBI BioProject number (PRJNA1177890) under the accession numbers CP173228, CP173229, CP173230, CP173231, CP173232, CP173233, CP173234, CP173236, JBJBHD000000000, JBJBHC000000000, JBJBHF000000000, and JBJBHE000000000 (Table 1). The SRA accession numbers for Illumina and ONT raw reads of each isolate are listed in Table 1.
